# Tocotrienols: A Family of Molecules with Specific Biological Activities

**DOI:** 10.3390/antiox6040093

**Published:** 2017-11-18

**Authors:** Raffaella Comitato, Roberto Ambra, Fabio Virgili

**Affiliations:** Council for Agricultural Research and Economics, Research Centre for Food and Nutrition (CREA-AN) via Ardeatina 546, 00178 Rome, Italy; raffaella.comitato@crea.gov.it (R.C.); roberto.ambra@crea.gov.it (R.A.)

**Keywords:** tocopherols, tocotrienols, estrogen receptors, endoplasmic reticulum stress, neuroprotection

## Abstract

Vitamin E is a generic term frequently used to group together eight different molecules, namely: α-, β-, γ- and δ-tocopherol and the corresponding tocotrienols. The term tocopherol and eventually Vitamin E and its related activity was originally based on the capacity of countering foetal re-absorption in deficient rodents or the development of encephalomalacia in chickens. In humans, Vitamin E activity is generally considered to be solely related to the antioxidant properties of the tocolic chemical structure. In recent years, several reports have shown that specific activities exist for each different tocotrienol form. In this short review, tocotrienol ability to inhibit cancer cell growth and induce apoptosis thanks to specific mechanisms, not shared by tocopherols, such as the binding to Estrogen Receptor-β (ERβ) and the triggering of endoplasmic reticulum (EndoR) stress will be described. The neuroprotective activity will also be presented and discussed. We propose that available studies strongly indicate that specific forms of tocotrienols have a distinct mechanism and biological activity, significantly different from tocopherol and more specifically from α-tocopherol. We therefore suggest not pooling them together within the broad term “Vitamin E” on solely the basis of their putative antioxidant properties. This option implies obvious consequences in the assessment of dietary Vitamin E adequacy and, probably more importantly, on the possibility of evaluating a separate biological variable, determinant in the relationship between diet and health.

## 1. Introduction

The term “natural Vitamin E” is commonly used to group together eight different molecules, namely α-, β-, γ- and δ-tocopherol and the corresponding tocotrienols. The original description of Vitamin E is: “a fat soluble vitamin that inhibits oxidative destruction of biological membranes and is necessary for fertility and to prevent hemolysis in rats and muscle dystrophy in poultry” [1]. If we held that this definition is biologically true and descriptive of the vitamin functions, we must face the evidence that, with the exception of a “generic” putative antioxidant activity in protecting biological membranes from peroxidation, in comparison to α-tocopherol, β-, γ- and δ-tocopherol and all tocotrienols have very low (if any) biological activity in the fetal re-absorption test. Their relative efficiency, in fact, ranges from zero to about 40% for β-tocopherol, which is the only alternative form significantly active in this test [1]. Accordingly, the units definition provided by the United States Pharmacopoeia of the different Vitamin E analogues is standardized on the efficiency of α-tocopherol, whereas β-, γ- and δ-tocopherol and the tocotrienols are much less active (either inactive) in this assay [2].

If we agree in considering that the only biological function of Vitamin E is acting as an “antioxidant”, the inclusion of tocotrienols in the Vitamin E family should not be questioned but, accordingly, we should include in the “Vitamin E family” several other molecules displaying a “lipid peroxidation chain breaking activity” within biological membranes. Conversely, if we accept that the biological role of α-tocopherol is more complex and goes beyond its activity as a membrane antioxidant, we must accept that this role is not shared by other tocols, and solely assign the term Vitamin E to α-tocopherol.

In fact, it is possible to describe similarities and differences between the members of the Vitamin E family. Dietary α-tocopherol, non-α-tocopherols and tocotrienols are all absorbed by intestinal cells by passive diffusion; and receptor-mediated transport and delivered to the lymph and then to the liver via chylomicron [3]. However, the efficiency of absorption is not the same, being apparently higher for tocotrienols than for α-tocopherol. This latter has been reported to significantly inhibit the cellular uptake of δ-tocotrienol (and probably of other tocotrienols), at least in endothelial cells by a still unknown mechanism [4].

Once in the liver, α-tocopherol is immediately transferred to the α-tocopherol transfer protein (α-TTP) (see below) and further disposed to the peripheral tissues after the incorporation into VLDL/LDL/HDL [3]. If not delivered to peripheral tissues, α-tocopherol, non-α-tocopherols and tocotrienols are metabolized by phase I and II enzymes and excreted as glucuronide or sulfate [3], but tocotrienols have been reported to be degraded to a larger extent than their counterparts with saturated side chains. The significant quantitative differences in the metabolism between each tocopherol and between tocotrienols and tocopherols that have been reported in vitro, suggest that similar differences may exist also in vivo [5].

Very importantly, the biological activity of Vitamin E is highly dependent upon regulatory mechanisms exerted by the intracellular α-TTP that enrich the plasma with α-tocopherol while non-α-tocopherols molecules are directed to metabolism. α-TTP specifically recognizes α-tocopherol by the three methyl groups on the chromanol ring, by the hydroxyl group on the chromanol ring and the structure and orientation of the phytyl side chain. Thanks to these multiple “recognition mechanisms”, α-TPP preferably and efficiently only binds α-tocopherol while all the other Vitamin E forms display a very low or null binding activity [6,7]. As the result of this specificity, only α-tocopherol, once taken up from the liver from dietary derived chilomicrons, can be released to peripheral extrahepatic tissues via lipoprotein VLDL trafficking, while the other tocopherols and the tocotrienols are rapidly metabolized by phase I and phase II enzymes and finally excreted in the bile, feces or urine [3]. Interestingly, a linear relationship between the relative affinity and the known biological activity obtained from the rat resorption-gestation assay exists, clearly indicating that the ability to bind to α-TTP is critical to determine the biological activity. However, it has been demonstrated that long oral supplementation of tocotrienol to mice and rats results in the delivery to vital organs including the brain, liver, heart, skin, lungs, adipose tissue, and whole blood independently of α-TTP expression [8,9]. These findings strongly demonstrate the existence of TTP-independent, still partially unknown, mechanisms of transport for oral tocotrienol. Moreover, the presence of α-tocopherol leads to decreased binding and to an acceleration of metabolism of non-α-tocopherols and tocotrienols [9].

Significant differences also exist in plasma concentration of α-tocopherol and tocotrienols. The administration of high doses (750 mg and 1000 mg) of a tocotrienol mixture from Annatto (*Bixa orellana*), results in a maximum plasma concentration levels at 3–4 h for all isomers, while α-tocopherol is reported to peak at 6 h, suggesting a different distribution mechanism [10]. Even though γ- and δ-tocotrienols have been probably studied more in the detail, α-tocotrienol has been found as the most abundant form circulating in plasma, chilomicrons, LDL, and HDL after the dietary supplementation with a mixture from palm (*Elaeis guineensis)* oil, also containing α-tocopherol. According to the study mentioned above [10], concentrations are in the order of µM and peak at less than 5 h for γ- and δ-tocotrienol and at 6 h for α-tocotrienol [11].

**Figure 1:**
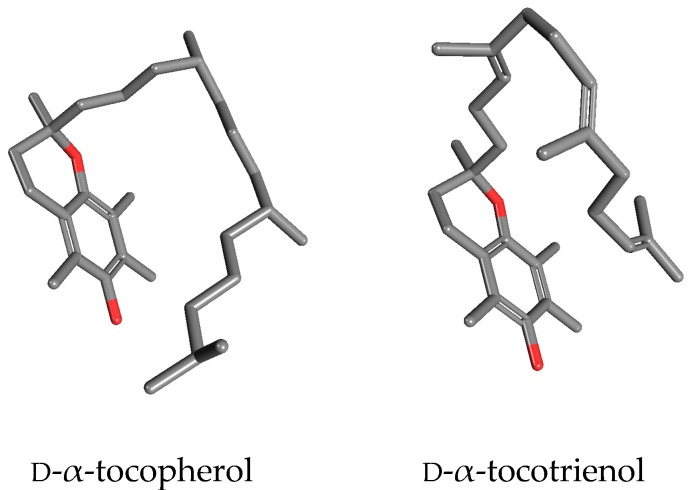
Structural differences between α-tocotrienol and α-tocopherol (from https://pubchem.ncbi.nlm.nih.gov/).

Overall, in conclusion, it is quite surprising that α-, β-, γ- and δ-tocotrienol are still pooled together with tocopherols in spite of a significantly different steric hindrance due to the unsaturated phytyl tail, and of several evidences indicating that they have a distinct metabolism and different routes of tissue delivery and storage [12]. All these evidences suggest that tocotrienol should be a candidate to play a different biological role than α-tocopherol [13]. A “side-to side” visualization of d-α-tocopherol and d-α-tocotrienols (Figure 1) highlights the evident differences between these two molecules at the tridimensional level that are frequently ignored in a two-dimensional, planar visualization. A dynamic view of these two molecules would further underline their different spatial seizures, due to the presence of two *trans* double bonds in the phytyl tail of tocotrienols limiting the rotational freedom of carbon-carbon bonds.

A plethora of papers, also published by high impact factor journals (including those by the authors) encourage a kind of confusion. In fact, the term Vitamin E is frequently introduced as (e.g.,): “…*Tocotrienols and tocopherols are natural forms of the Vitamin E family…*”, even though it is always admitted that (e.g.,) “*… Vitamin E deficiency syndromes cannot be prevented by supplying non-α-tocopherols or tocotrienols…*” and that (e.g.,) “*Although all natural forms of Vitamin E display potent antioxidant activity, the activity of tocotrienols is mediated independently of their antioxidant activity*..” and also: *“… current studies of the biological functions of Vitamin E indicate that members in the Vitamin E family possess unique biological functions often not shared by other family members*”.

Starting from the “contradictions” described above, this review focuses on some of the most evident specific activities reported for tocotrienols, not shared by α-tocopherol or by other tocopherols, namely the estrogen receptor-β (ERβ) binding activity and endoplasmic reticulum (EndoR) response activation both leading to a pro-apoptotic cellular response by γ- and δ-tocotrienol. The specificity of α-tocotrienol in protecting neuronal cells after ischemia will also be presented and discussed together with other tocotrienol-specific mechanisms on cell functions and survival.

## 2. Tocotrienols as Ligands of ERβ

Interestingly, one of the first studies reporting the antiproliferative/pro-apoptotic effects of tocotrienols, excluded the possibility of a mechanism related to the binding to estrogen receptors [14]. This early paper reported a study that was conducted somehow before the discovery and characterization of the β form of ERs [15]. The experimental design was in fact based on the assumption that the human breast cancer cell line estrogen-independent (MDA-MB-231) was void of any ER form and utilized to compare the effects of tocotrienols on the estrogen responsive breast cancer cell line, MCF7. Conversely, MDA cells have been eventually demonstrated to express a functional β form, while MCF7 express both α- and β-ER. In this study, authors observed that a tocotrienol-rich fraction (TRF) of palm (*Elaeis guineensis*) oil, containing α-tocopherol and α-, γ- and δ-tocotrienol, inhibited MCF7 cells growth in both the presence and absence of estradiol with a nonlinear dose-response. MDA-MB-231 cells were also inhibited by TRF but with a linear dose-response. In the same study, the authors reported, after fractionation of TRF, γ- and δ-tocotrienols containing fractions were the most inhibitory ones. On the other hand, and according to the matter of the present paper, α-tocopherol had no effect on the growth rate of both cell lines. The authors ruled out the contribution of ER activity on the basis that TRF (and therefore tocotrienols) did not affect the expression of a gene, the breast cancer estrogen inducible sequence-trefoil factor 1 (pS2-TFF1), known to be driven by estradiol due to the presence of an ERα responsive elements in its promoter. The original conclusion has been reconsidered under the light of an “*omic*” approach utilized in subsequent studies performed by our laboratory. In a first study [16], utilizing cultured cells as an experimental model, we performed a cDNA array analysis of cancer-related gene expression in estrogen-dependent (MCF-7) and estrogen-independent (MDA-MB-231) human breast cancer cells. In this study, we utilized the best transcriptomic platform available at that time (1200 gene) that allowed us to conclude that the supplementation tocotrienol rich fraction form palm oil (TRF) was associated to the modulation of a number of genes encoding for proteins involved in cell cycle and therefore to inhibitory effects on cell growth and differentiation of the tumor cell lines. Previous studies had already demonstrated that α-tocopherol, the only tocopherol form present in TRF, had no effect on the induction of apoptosis in both cell lines, the only one having some pro-apoptotic activity being δ-tocopherol [17].

In a second study, based on a more sophisticated in vivo model [18], MCF-7 breast cancer cells were injected into athymic nude mice also fed with TRF. At the end of 20 wk dietary treatment there was a significant delay in the onset, incidence, and size of the tumors in nude mice supplemented with TRF compared with the controls. In addition, a cDNA array technique providing the differential expression of 1200 genes was performed on excised tumor tissues and, in agreement with the study performed on cultured cells, a significant number of genes was affected by TRF treatment. According to a “gene-ontology” analysis we identified a set of genes involved in the regulation of immune response and in the functional class of intracellular transducers/effectors/modulators. Data obtained in the course of these studies, further interrogated in silico provided a consistent hypothesis for a direct interaction of tocotrienols with estrogen pathway. In silico docking analysis, and in vitro binding-displacement test to purified ER protein demonstrated a high affinity of tocotrienols for ERβ but not for ERα. We also demonstrated that in ERβ-containing MDA-MB-231 breast cancer cells, tocotrienols, but not α-tocopherol, increase ERβ translocation into the nucleus and the expression of a spectrum of pro-apoptotic estrogen-responsive genes such as the Macrophage-inhibiting Cytokine-1 (MIC-1), the Early Growth Response-1 (EGR-1) and Cathepsin-D and accompanied by typically apoptotic-like alterations of cell morphology, DNA fragmentation, and caspase-3 activation [19]. These results have been corroborated and completed by a second study conducted on MCF-7 breast cancer cell, expressing both ERα and ERβ [20]. Furthermore, in this cell line, treatment with TRF and in particular with γ-tocotrienol, but not α-tocopherol, was associated with ERβ nuclear translocation and ER-dependent genes expression (MIC-1, EGR-1 and Cathepsin-D). At same time, the treatment induced a very evident inhibition of ERα activity finally leading to DNA fragmentation and apoptosis [20]. cDNA-array data obtained within this study also suggested the presence of an alternative pathway activated by γ- and δ-tocotrienols that will be presented below.

It is well known that ERβ activation can produce different cellular outcomes according to the balance between the “non genomic” signaling triggered by ERα and ERβ and other specific characteristics of cellular and tissue environment [21,22]. Accordingly, Nakaso and coworkers have more recently reported a cytoprotective, rather than pro-apoptotic effect of γ- and δ-tocotrienol in SH-SY5Y neuroblastoma cells, a cellular model addressing the pathogenesis of human Parkinson’s disease [23]. In agreement with our original studies, this study confirmed that purified γ- and δ-tocotrienol bind to ERβ in vitro and that γ- and δ-tocotrienol were cytoprotective against Parkinson’s disease-related toxicities such as 1-methyl-4-phenylpyridinium ion (MPP^+^) thanks to a marked activation of the phosphoinositide-3-kinase/serine/threonine kinase-1 (PI3K/Akt) signaling pathway downstream to ERβ binding. The pivotal and functional role of γ- and δ-tocotrienol binding to ERβ was confirmed by ERβ silencing that was associated to the abrogation of cytoprotection and Akt phosphorylation [23].

The same authors also reported that the silencing of caveolin-1 and/or caveolin-2, candidates for the early events of signal transduction, prevented the cytoprotective effects of γ- and δ-tocotrienol, but not Akt phosphorylation. In agreement with us, the authors conclude that tocotrienols, in particular γ- and δ-tocotrienol, have distinct biological activities, unrelated to their putative antioxidant capacity, and that they can exert a specific neuro-protective activity mediated by ERβ binding and PI3K/Akt signaling activation [23].

Remarkably, one early study reported that tocotrienols induce apoptosis and a significant delay in cell growth in normal mammary cells obtained from midpregnant BALB/c mice [24]. However, neither the same authors, nor other research groups, have eventually confirmed these observations. Conversely, the same authors more recently, reported that γ-tocotrienol has a potent antiproliferative and cytotoxic effects by autophagy in MCF-7 and MDA-MD-231 cancer cell [25]. In contrast to the reports of Nakaso and coworkers mentioned above [23], γ-tocotrienol is reported to induce a reduction in PI3K/Akt/ mechanistic target of rapamycin kinase (mTOR) signaling and a corresponding increase in the Bax/Bcl-2 ratio, cleaved caspase-3, and cleaved poly (ADP-ribose) polymerase (PARP) levels in these cancer cell lines. These events suggest that γ-tocotrienol-induced autophagy may be involved in the initiation of apoptosis. In contrast, the same treatment was not found to increase autophagy marker expression in immortalized mouse (CL-S1) and human (MCF-10 A) normal mammary epithelial cell lines [25].

It seems therefore possible to speculate that γ-tocotrienol induces opposite effects in cancer and normal (or pseudo-normal) cells in agreement with the established perturbation of PI3K/Akt/mTOR signaling pathway in cancer cells [26]. Similarly, a specific effect of tocotrienols on Bax/Bcl2 mediated apoptosis has been also reported in prostate tumorigenesis in the transgenic adenocarcinoma mouse prostate (TRAMP) mouse model. In this study, a dietary supplementation with of a tocotrienol mixture induced a decrease in the levels of high-grade neoplastic lesions associated with an increased expression of proapoptotic proteins BAD a Bcl2 antagonist of cell death and cleaved caspase-3 and cell cycle regulatory proteins cyclin dependent kinase inhibitors p21 and p27 [27].

## 3. Tocotrienols as Inducers of Apoptosis via Endoplasmic Reticulum (EndoR) Stress

The first report of an EndoR stress mediated apoptosis by tocotrienols dates back about 10 years. Wali and coworkers [28] tested the effect of γ-tocotrienol in highly metastatic +SA rodent mammary epithelial cells. This study identified that 15–40 µM γ-tocotrienol induces a dose dependent apoptotic response paralleled by an increase of poly (ADP-ribose) polymerase (PARP)-cleavage and activation of a pathway distinctive of EndoR stress response, the protein kinase-like endoplasmic reticulum kinase/eukaryotic translational initiation factor/activating transcription factor 4 (PERK/eIF2α/ATF-4). γ-tocotrienol treatment also caused a large increase of key components of EndoR stress mediated apoptosis, tribbles 3 (TRB3) and C/emopamil binding protein (EBP) homologous protein (CHOP). The silencing of this latter gene significantly quenched γ-tocotrienol-induced PARP-cleavage and TRB3 expression. The same study [28] also reports that γ-tocotrienol treatment was associated to a decrease of full-length caspase-12 levels, indicating an activation of caspase-12 cleavage.

Park and collaborators have later confirmed these observations in a syngeneic mouse mammary tumor model and in cultured human breast cancer cells [29]. In the animal study, after the subcutaneous implantation of 66cl-4-GFP murine mammary tumor cells, female BALB/c mice were fed a diet enriched with purified α- and γ-tocotrienol to approximately provide 0.625 mg of each tocotrienol/mouse/day. The authors excluded a possible interference from Vitamin E supplementation, by adjusting Vitamin E in the diet with 30 IU/kg diet of dl-α-tocopheryl acetate to fully meet the animal’s requirement. Dietary γ-tocotrienol suppressed tumor growth by inhibiting cell proliferation and inducing apoptosis. In fact, tumors excised from the γ-tocotrienol fed animals had significant more TUNEL (terminal deoxynucleotidyl transferase mediated nick end labeling assay) and less Ki-67 (a biomarker for cell division) positive cells (386% and 55%, respectively) compared to the animal fed the basal diet group. The same study reports no effects on tumor vascularization, and that the feeding with α-tocotrienol supplementation was not associated to comparable activities potential, at the tested dose. When utilizing cultured cells (MDA-MB-231 and MCF-7 human breast cancer cells and 66cl-4-GFP murine mammary tumor cells) the authors could confirm that α-, γ- and δ-tocotrienols induce apoptosis, independently of cell estrogen receptors’ expression profile [29]. γ-tocotrienol was the most active form and was studied in deeper detail. The authors observed a significant activation of c-Jun NH(2)-terminal kinase (JNK) and p38-mitogen activated kinase-like protein (MAPK) accompanied by the upregulation of the expression death receptor 5 (DR5) and C/EBP homologous protein (CHOP), an endoplasmic reticulum (EndoR) stress marker, these events finally leading to the PARP, caspase-8, -9, and -3 cleavage. The silencing of JNK or p38 MAPK was associated to a reduced increase of DR5 and CHOP and partially inhibiting apoptosis. α-tocotrienol did not reduce tumor burden in vivo, but had inhibitory effects on colony formation in all three cell lines at relatively high concentrations in comparison to γ- and δ-tocotrienols, corroborating the evidence that the observed effects are strongly specific.

A further indication for the involvement of EndoR stress in tocotrienol-induced apoptosis was provided by Patacsil and coworkers [30] in MDA-MB 231 and MCF-7 breast cancer cells. This study demonstrates that 40 µM γ-tocotrienol induces PARP cleavage and caspase-7 activation in MCF-7 cells, accompanied by alterations in the expression of genes involved in cell growth and proliferation, cell death, cell cycle, cellular development, cellular movement and gene regulation. Among the categories studied by means of in silico Pathway Analysis the paper reports the modulation of signal transduction mediated by NRF-2-mediated “hormetic” oxidative stress response, transforming Growth Factor-β (TGF-β) signaling and EndoR stress response. Moreover, in agreement with other studies conducted on the same cellular models [31], the same study reports that MCF-7 and MDA-MB 231 cells respond to γ-tocotrienol by inducing the activation of PERK and pIRE1α pathways. The strong up-regulation of the Activating transcription factor 3 (ATF3) (16.8-fold) associated to EndoR stress and the abrogation of the apoptotic response after ATF3 silencing suggests this protein as a potential molecular target for γ-tocotrienol in breast cancer cells.

The specific activity of γ-tocotrienol on CHOP expression and EndoR stress has been also reported in several human malignant mesothelioma H2052 (sarcomatoid), H28 (epithelioid), H2452 (bi-phasic), and MSTO-211H (biphasic). In these cells, statins (atorvastatin and simvastatin) and 20 µM γ-tocotrienol have been observed to have synergistic effect on cell growth inhibition and acting through the inhibition of mevalonate pathway [32]. The authors report that, in H2052 and MSTO-211H cells, the treatment with γ-tocotrienol alone was sufficient to induce a significant increase of the expression of the endoplasmic reticulum stress markers CHOP and glucose regulated protein-78 (GRP-78). Conversely, the intrinsic apoptotic marker, caspase 3 activation, was induced only in the presence of statins. It is well known that the Bcl-2 family plays a pivotal role in apoptosis, either as an activator (through Bax and Bak) or as an inhibitor (Bcl-2 and Bcl-xL), Bcl-2 to Bax ratio is recognized as a key factor in the regulation of the apoptotic process or cell death [33]. According to this notion, this study also considered the possibility that γ-tocotrienol could affect Bax to Bcl2 ratio. Consistently with a previous report indicating that γ-tocotrienol induced a mitochondrial disruption pathway without affecting Bax/Bcl-2 expression in human breast cancer MDA-MB-231 cells [34], the Bax/Bcl2 ratio was not affected by any of the treatments.

We have also studied the involvement of EndoR stress on the effects of δ-tocotrienol on the growth of two different lines of human melanoma cells, BLM and A375 [35]. In agreement to observations obtained utilizing different tumor lines, the treatment with 5–20 µM δ-tocotrienol had a significant proapoptotic effect on both cell lines, involving the intrinsic apoptosis pathway. Very importantly, we observed no effect on the viability of normal human melanocytes. δ-tocotrienol effects were associated to the activation of the PERK/p-eIF2α/ATF4/CHOP, Inositol-requiring enzyme-1α (IRE1α) and caspase-4 strongly suggesting the involvement of EndoR stress upstream to the apoptotic response. We confirmed this hypothesis observing the quenching of the apoptotic response after a treatment with Salubrinal, an inhibitor of the EndoR stress. In the same study, in disagreement with observations reported by others [32,34], but in agreement with the observations by Barve and coworkers [27], we observed cytochrome c release from mitochondria, indicating a disruption of the mitochondrial outer membrane potential, associated to a significant increase of Bax/Bcl2 ratio. In vivo experiments performed in nude mice bearing A375 cells xenografts confirmed that a supplementation with dietary δ-tocotrienol (100 mg/kg daily, 5 days/week) up to 35 days, results in a reduced tumor volume and tumor mass and in significant delay of tumor progression [35].

A final confirmation of the ability of specific tocotrienol forms to activate EndoR stress is reported in a very recent study from our laboratory [31]. We reported a series of experiments based on the analysis of transcriptomic data obtained within our previous studies [18,19]. These data, interrogated by different bio-informatics tools, suggested the existence of an alternative pathway, activated by specific tocotrienols forms and leading to apoptosis, also in tumor cells not expressing ERs. This hypothesis was, in fact, confirmed utilizing HeLa cells, a line of human cervical cancer cells void of any canonical ER form. Once synchronized and treated either with the tocotrienol-rich fraction (α, γ and δ form) from palm oil (10–20 μg/mL) or with purified α-, γ- and δ-tocotrienol (5–20 μg/mL), HeLa cells underwent apoptosis which was accompanied by a significant expression of caspase 8, caspase 10 and caspase 12. Following the interrogation of additional data obtained from transcriptomic platforms, we considered the hypothesis that the administration of γ- and δ-tocotrienol could induce a release of Ca^2+^ from the EndoR. Accordingly, in living cells, we observed a significant activation of Ca-dependent signals. This event was followed by the expression and activation of IRE-1α and by the splicing and activation of X-box binding protein 1 (XBP-1), another molecule involved in the unfolded protein response, the core pathway coping with EndoR stress in eukaryotic cells, finally leading to apoptosis. Very importantly, and not very commonly indeed, our study considered α-tocopherol as a negative control, confirming that only γ- and δ-tocotrienol, not the proper Vitamin E d-α-tocopherol, are responsible for the observed effects. In the same paper [31], addressing the molecular mechanism underlying tocotrienols’ activity, we wanted to speculate about the possible presence of a putative (orphan) receptor, possibly located at the level of the cellular membrane and able to accept tocotrienols and other estrogen mimetics as selective ligands. We based this speculation on the observation that the treatment with the specific ER inhibitor ICI-182,780 weakens the effects of tocotrienols on the upregulation of pro-apoptotic genes and the apoptotic response in cells lacking ERs. According to the chemo-physical characteristics of tocotrienols, the candidate downstream target(s) of the activity of this receptor could reasonably be located at the level of EndoR. The activation of this hypothetical (orphan) receptor would sequentially trigger EndoR stress, IRE-1 activation and XBP-1 splicing, finally inducing apoptosis.

The ability of γ-tocotrienol to induce apoptosis through activation of both the intrinsic and extrinsic pathway has been also confirmed in Jurkat cells, a human T-cell lymphoma [36]. In this study, γ-tocotrienol but not α-tocotrienol inhibited proliferation and induced apoptosis in this cell line in a dose dependent manner. The administration of 10–50 µM γ-tocotrienol resulted in elevated mitochondrial ROS production, JNK activation and suppression of extracellular regulated MAP kinase (ERK) and p38-MAPK. In agreement with our observations [31], γ-tocotrienol induced intracellular calcium release and, in agreement with others [27] a loss of mitochondrial membrane potential and cytochrome c release. These changes were found to be associated with an increase Bax/Bcl-xL expression rate and to an increased expression of Fas and FasL. Similarly to other studies [24,25,35], γ-tocotrienol had no effects on normal human peripheral blood mononuclear cells suggesting a specific cytotoxicity towards transformed lymphoma cells [36].

γ-tocotrienol has also been demonstrated to induce paraptosis, a type of caspase independent programmed cell death, morphologically distinct from apoptosis, in that it displays cytoplasmic vacuolation and lacks of the typical apoptotic morphology. Zhang and collaborators [37] focused on the effects of δ-tocotrienol on human colon cancer SW620 cells and observed that δ-tocotrienol inhibits proliferation in a dose-dependent manner, correlated with cytoplasmic vacuolation possibly resulting from welling and fusion of mitochondria and/or EndoR. No changes in caspase 3 activation and other morphological (blebbing) or molecular markers of apoptosis were observed. The authors also report that δ-tocotrienol treatment (10 to 40 µM) was associated to a reduced β-catenin and wnt-1 expression and to lower cyclin D1, c-jun and matrix metallopeptidase 7 (MMP-7) protein levels, indicating the triggering of paraptosis-like cell death, with the suppression of the Wnt signaling pathway. The same authors have more recently reported similar results after the treatment with γ-tocotrienol both in SW620 and HCT-8 cells, another type of human ileocecal colorectal adenocarcinoma also associated to the suppression of Wnt signaling pathway [38].

## 4. Neuroprotection and Lipoxygenase Inhibition by α-Tocotrienol

While γ- and δ-tocotrienol have been demonstrated to induce specific responses in cancer cells with little or no effects on normal cells, α-tocotrienol has been frequently reported to play a protective activity on normal neuronal cells and, in general, on central nervous system. A study by Fukui and collaborators [39] utilized neuro2a cells, a line obtained from a spontaneous neuroblastoma in an albino strain A mouse frequently utilized as a model to study neurite outgrowth, neurotoxicity, Alzheimer disease and in general for neuronal tumourigenicity studies. This study, even appearing somehow an oversimplification in comparison with other studies based on a more complex experimental design, demonstrated that 5 µM α-tocotrienol counters the effects of the water-soluble free radical generator 2,2′-azobis(2-methylpropionamide) dihydrochloride (AAPH) on neurite degeneration and dynamics.

More specific research, directly targeting the protective effects of α-tocotrienol against neurodegeneration, has been conducted by the group of Sen and collaborators [40]. These authors originally reported the ability of nanomolar concentrations of α-tocotrienol, but not α-tocopherol to block glutamate-induced cell death. Glutamate is one of the most important neurotransmitters but, at high concentrations, it induces a rise of intracellular Ca^2+^ and mitochondrial dysfunction followed by cell death [41]. α-tocotrienol inhibits cell death by suppressing the activation of c-Src kinase and ERK phosphorylation in HT4, an embryonal carcinoma from metastatic lung with neural characteristics and expressing Simian vacuolating polyoma virus 40 (SV40) [42]. Very interestingly, the concentrations utilized in this study were 4–10-fold lower than levels detected in plasma of supplemented humans, indicating that α-tocotrienol regulates a specific signal transduction pathway insensitive to comparable concentrations of tocopherol, clearly suggesting that this activity is completely independent of its antioxidant capacity.

In a following study [43], the same group demonstrated that, in the same tumor cell line (HT4) and in immature primary cortical neurons, glutamate toxicity is mediated by the activation of neuronal 12-lipoxygenase that precede the production of peroxides, increased influx of Ca^2+^ and cell death. At lower physiologically achievable concentrations (nanomolar), α-tocotrienol displayed potent neuroprotective properties in both cell lines challenged by glutamate apparently interacting with 12-lipoxygenase, suppressing arachidonic acid metabolism. This observation was confirmed in vitro, by testing the activity of a purified enzyme in the presence of α-tocotrienol and by an in silico docking study suggesting that α-tocotrienol hampers the access of lipoxygenase substrate, to the enzyme catalytic site [43]. Even though a 12-lipoxygenase inhibiting activity has been reported also for α-tocopherol [44], the IC50 was in the order of µM, which is an order of magnitude higher than that reported for α-tocotrienol and, therefore, possibly due to a different mechanism.

The same group has further investigated the neuroprotective activity of α-tocotrienol in a sophisticated model based on single neuron microinjection technique on HT4 cells. The same study also reports the effects of dietary supplementation of α-tocotrienol in 12-lipoxigenase deficient mice undergoing surgically induced stroke and in spontaneously hypertensive rats [45]. The authors observed that very low quantities (sub-attomole) of α-tocotrienol, but not of α-tocopherol, protected isolated neurons from glutamate challenge. In agreement with the observations of a rapid 12-lipoxygenase tyrosine phosphorylation catalyzed by c-Src, lipoxygenase-deficient mice were more resistant to stroke-induced brain injury than their wild-type controls. Oral supplementation of α-tocotrienol to spontaneously hypertensive rats was associated to increased levels in the brain and to a protection against stroke-induced injury, associated with lower c-Src activation and 12-lipoxygenase phosphorylation at the stroke site.

In a study that followed [46], the same group addressed the comparison between the antioxidant-independent and -dependent neuroprotective properties of α-tocotrienol in HT4 cells cytotoxicity elicited by homocysteic acid and linoleic acid, respectively. Homocysteic acid administration was associated to a significant neurodegeneration presenting features similar to those previously observed in glutamate-induced neurotoxicity, in particular, the activation of c-Src and 12-lipoxygenase phosphorylation as early events. The administration of both homocysteic and linoleic acid was associated to an increased ratio of oxidized to reduced glutathione and to the triggering of an EndoR stress, as indicated by raised intracellular Ca^2+^ concentration and altered mitochondrial membrane potential eventually followed by cell death [46]. As the oxidative stress is considered a late event in apoptosis induced by homocysteic acid, the antioxidant component of α-tocotrienol protection was verified by inducing oxidative stress and cell death by linoleic acid. In all cases, the presence of nanomolar concentrations of α-tocotrienol, but not of α-tocopherol, was significantly protective. The authors concluded that α-tocotrienol protection against neurotoxicity is attributable to a combination of an antioxidant-independent and to antioxidant-dependent mechanisms.

The neuroprotective activity tocotrienols and, in particular, of α-tocotrienol has been confirmed by Osakada and coworkers [47]. In their study, the authors observed that a tocotrienol mixture from palm oil, further purified in order to eliminate α-tocopherol, purified α-, γ- and δ-tocotrienol (0.1–10 µM) significantly protects primary neuronal cells from rat striatum challenged by different pro-oxidants. Namely, hydrogen peroxide, a superoxide generating molecule (paraquat), nitric oxide donors (*S*-nitrosocysteine and 3-morpholinosydnonimine) and an inhibitor of glutathione synthesis l-buthionine-[*S*,*R*]-sulfoximine were utilized. In the same paper, only α-tocotrienol, but not γ- and δ-tocotrienol prevented the apoptosis induced by a protein kinase inhibitor, staurosporine, suggesting a specific mechanism underlying α-tocotrienol activity, totally independent of its putative antioxidant capacity. Conversely, α-tocopherol was ineffective in all cases, corroborating the evidence of a specific tocotrienol activity unrelated to their nucleophilic, antioxidant, properties.

It is worth noting that Wang and coworkers have elegantly demonstrated that the arylating oxidative product of γ-tocopherol, γ-tocopherol quinone, but not the non-arylating α-tocopherol quinone, induces EndoR stress affecting activation of PERK/CHOP proteins in N2A neuroblastoma cells [48]. As mentioned above, γ- and δ-tocotrienol have been also reported to affect this pathway in melanoma [35], mesothelioma H2052 and MSTO-211H cells [32], while our recent study indicated that EndoR stress induced by tocotrienols was mediated by IRE1 activation and not by PERK, suggesting a separate mechanism of action [31]. However, the paper by Wang and collaborators [48] clearly identifies the arylating activity of γ-tocopherol quinone as the mechanism underlying the activation of EndoR stress, while other investigations and in particular our study [31], did not take this event into consideration, on the basis of the indications of a receptor-mediated mechanism.

Finally, it is possible that the relative intracellular concentration and the specific localization of α-tocopherol and γ- and δ-tocotrienol can affect the cell response, eventually determining the final outcome. At present, very few studies [45] addressed this important issue and more investigation is surely warranted.

## 5. Other Tocotrienol-Triggered Cellular Responses

Besides ERβ activation and EndoR stress mediated apoptosis, γ-tocotrienol has also recently been observed to significantly alter sphingolipids composition in various types of cancer cells such as Human colon HCT-116, pancreatic PANC-1 and breast MCF-7 [49]. In particular, the authors observed a rapid elevation of dihydrosphingosine and dihydroceramides associated with increased cellular stress, phosphorylation of the mitogen-activated protein kinase, JNK and apoptosis. The inhibition of the de novo synthesis of sphingolipids was associated to a parallel inhibition of γ-tocotrienol-induced apoptosis and autophagy. γ-tocotrienol was demonstrated to inhibit dihydroceramide desaturase (DEGS) activity but not its protein expression or de novo synthesis of sphingolipids. The increase of dihydroceramides paralleled by a relative decrease of ceramides coincides with the induction of apoptosis and autophagy. Overall, the authors conclude that γ-tocotrienol inhibits ceramide desaturase activity inducing an early elevation of dihydro-sphingolipids and late increase of saturated ceramide suggesting a role of these sphingolipid in tocotrienol-induced cell stress and death [49].

According to the evidence that several chemopreventive agents negatively affect cell growth by targeting p53 pathway, Agarwal and coworkers [50], utilized the human colon carcinoma RKO cell line, to investigate the effects of a TRF on the components of p53 signaling network. The authors report that the treatment with TRF results in a dose- and time- dependent inhibition of growth and colony formation associated to the induction of cyclin dependent kinase inhibitor 1A (WAF1)/p21, independent of cell cycle regulation and is transcriptionally upregulated in p53 dependent fashion.

In agreement with observations from our group and by others [27,35], but in disagreement with other reports [32,34], Agarwal and collaborators [50] reported that TRF induces the release of cytochrome c and induction of apoptotic protease-activating factor-1 upon a significant alteration of Bax/Bcl2 ratio. The altered ratio between the members of the Bax family triggered in turn by the activation of initiator caspase-9 followed by activation of effector caspase-3 finally lead to apoptosis characterized by chromatin condensation, DNA fragmentation and shrinkage of cell membrane.

Other biological activities have been reported specifically for γ- and δ-tocotrienol. A recent paper by Wong and coworkers [51] reports that the supplementation with 85 mg/kg/day γ- and δ-tocotrienol, but not with α-tocotrienol and α-tocopherol, improves cardiovascular functions in rats fed for 16 weeks a diet high in simple carbohydrates (fructose) and fats (beef tallow), in comparison to a corn starch based diet. In the same study [51], only δ-tocotrienol was associated with improved glucose tolerance, insulin sensitivity, lipid profile and abdominal adiposity. In the liver, these interventions reduced lipid accumulation, inflammatory infiltrates and plasma liver enzyme activities. In agreement with previous investigations [8,9], despite low or no detection of tocotrienols in plasma, the authors found detectable levels in heart, liver and adipose tissue confirming that chronic oral administration is associated to tocotrienols delivery and accumulation to these organs [51].

## 6. Conclusions

We have provided some references that strongly indicate that specific tocotrienols forms, and in particular α-, γ- and δ-, have biological activities distinct from that of tocopherols and in particular of d-α-tocopherol, the only molecule that, in our opinion should be considered as “Vitamin E”.

In this review, we focused our interest on the ability of γ- and δ-tocotrienol to induce cell growth arrest and apoptosis in different tumor cell types, by specific mechanisms apparently not shared by tocopherols. Even though other specific effects have been reported, the ability to act as “productive” ligands of ERβ and to trigger Ca^2+^ release from EndoR, seem the most evident features of δ- and γ-tocotrienol, eventually associated with the expression of pro-apoptotic genes, finally leading to apoptosis or paraptosis. Interestingly, the pathway associated to cell death depends, at least in part, on the tumor type and pro-apoptotic effects have not been reported in non-tumor cells.

**Figure 2:**
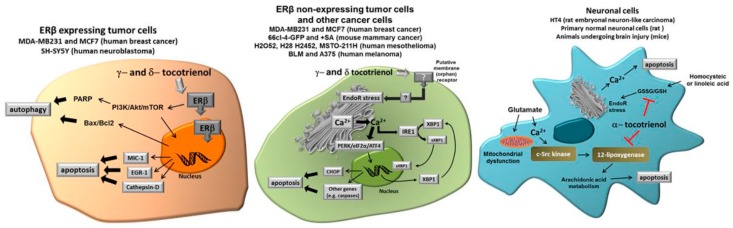
Different molecular targets of tocotrienols in the representative cell types we have considered in this review, namely cancer cells expressing Estrogen Receptor (ER), cancer cells not expressing ER and normal neuronal cells submitted to specific stressors such as glutamate or homocysteic acid. See text for more details. (Modified from [31]).

These observations not only suggest that specific forms of tocotrienols (in this case, δ- and γ-tocotrienol) have a specific capacity in inducing programmed death in tumor cells, but also that their specific activity occurs through diverse specific mechanisms, according to the cell type. Under this perspective, specific forms of tocotrienols and, in particular, δ-tocotrienol could be proposed as an expedient, potentially effective option for novel chemopreventive/therapeutic strategies in different types of solid tumors. On the other hand, α-tocotrienol displays a specific neuroprotective capacity due to its ability to inhibit 12-lipoxygenase, a key enzyme in the execution of cellular death induced by glutamate and by homocysteic acid. Taken together, current findings strongly indicate that tocotrienols may have a significant role in different specific pathological conditions, which are not necessarily affected by Vitamin E (α-tocopherol). Figure 2 shows the different molecular targets of tocotrienols in the representative cell types we have considered in this review, namely cancer cells expressing ER, cancer cells not expressing ER and normal neuronal cells submitted to specific stressors such as glutamate or homocysteic acid.

In spite of the relative abundance of studies dealing with the effects associated to the administration of tocotrienols, either in cultured cells or in experimental animals, very few studies considered the mechanism of action. In fact, very often, the terms “mechanism” and “effects” are utilized interchangeably, when they should refer to different concepts. In our opinion, the term mechanism should be solely utilized to describe the molecular events leading to the observed effects, such as (e.g.,) in the case of the tocotrienol binding to ERβ (mechanism) leading to the transcriptional activation of gene expression (effects). To our knowledge, our laboratory and few others [20,43] are the only ones that addressed this issue by the combination of in silico *docking* analysis, to build up the hypothesis of ER (either other nuclear receptors) binding or the inhibition of 12-lipoxygenase, respectively, eventually confirmed in vitro, in cultured cells and in animals. Further studies, dealing with the understanding of the molecular (mechanistic) basis underlying the observed effects of specific tocotrienol forms are surely warranted, in order to define the real limits of these molecules as a possible alternative for cancer treatment or co-adjuvant in cancer treatment.

Besides this, a major problem in the correct interpretation of some of studies reported herein is, in general, the absence of a treatment, either in cultured cells or in animals, with α-tocopherol. In fact, for instance, studies utilizing mixtures administered to cultured cells or to animals contained significant amounts of α-tocopherol. This experimental weakness make it unclear if the observed effects can be totally ascribed to tocotrienols or also to α-tocopherol, as several authors tend to claim. However, studies correctly performing this specific “negative” control published by our laboratory [19,20,31] and by others [29,40,42,43,45,46,47] provide indirect but solid indications about the differential ability of α-, γ- and δ-tocotrienol, but not α-tocopherols to trigger specific cellular response in different cell types.

## 7. Practical Consequences at Nutritional Level

If we accept that each tocotrienol form has a distinct, specific biological activity, different cellular targets and molecular mechanisms of action, significantly different from tocopherol and more specifically from α-tocopherol, we must necessarily stop pooling them together on solely the basis of their putative antioxidant properties. Consequently, tocotrienols should be excluded from the “Vitamin E family”. This option would probably imply some consequences in the assessment of dietary Vitamin E adequacy even though more studies are surely needed to investigate their relative body distribution and plasma levels in normal dietary conditions. More importantly, the distinction between Vitamin E (α-tocopherol) and specific non-α-tocopherol molecules will allow for investigations aiming to evaluate a separate biological variable determinant in the relationship between diet and health.

Finally, a system biology approach, accompanied by high throughput methodologies, appears an indispensable tool for a better understanding of the role of molecules of nutritional interest in human health and disease. Classical “hypothesis driven studies”, even when very well conducted and designed, may risk consolidating original weaknesses, possibly leading to a “*narrowing of perspective and from there false expectation*”.
